# Occlusion Break Surge and Anterior Chamber Stability in the Intraocular Environment of Modern Phacoemulsification: A Narrative Review

**DOI:** 10.3390/medicina62020298

**Published:** 2026-02-02

**Authors:** Hugo Scarfone, Emilia Carolina Rodríguez, Javier Diez, Ana Scarfone, Franco Scarfone

**Affiliations:** 1 Clínica de Ojos Tandil, Tandil B7000, Argentina; rodriguezemiliacarolina@gmail.com (E.C.R.); anitina.scarfone@gmail.com (A.S.); 2 Instituto de Física Arroyo Seco, Universidad Nacional del Centro de la Provincia de Buenos Aires, CIFICEN-CONICET-CICPBA, Tandil B7000, Argentina; diez.javier.a@gmail.com; 3 Clinica Charles, Buenos Aires C1116, Argentina; francoscarfone@gmail.com

**Keywords:** phacoemulsification, post-occlusion break surge, intraocular environment, anterior chamber stability, active fluidics, predictive fluidics, intraocular pressure, cataract surgery safety

## Abstract

Phacoemulsification is performed within a highly dynamic intraocular environment, in which fluid exchange, pressure regulation, and tissue biomechanics interact continuously. Although modern cataract surgery is considered safe and efficient, disruption of this delicate intraoperative microenvironment remains a major source of complications. Among fluidics-related events, post-occlusion break surge represents one of the most critical destabilizing factors of the anterior chamber. A surge occurs when the sudden release of an occluded aspiration port generates an abrupt pressure–volume imbalance that cannot be immediately compensated by infusion, leading to a transient collapse of the intraocular environment. This narrative review integrates current experimental and clinical evidence on the pathophysiology, quantification, and technological control of surge, framing it as a model of environmentally driven intraoperative stress. The evolution of phacoemulsification fluidics, from gravity-based systems to active, adaptive, and predictive platforms, is analyzed in relation to their ability to preserve a stable and physiologic intraocular environment. Comparative data from contemporary devices are reviewed, highlighting differences in surge volume, recovery time, and pressure restitution. Special emphasis is placed on the impact of surge on the microenvironments of both the anterior and posterior segments, including endothelial stress, capsular instability, vitreoretinal traction, and macular perfusion. Emerging strategies such as handpiece-integrated pressure sensors, predictive fluidics algorithms, intraoperative imaging, and artificial intelligence are reshaping environmental control during surgery. Despite substantial technological progress, the complete elimination of surge remains an unmet need. Continued innovation, standardized biomechanical models, and robust clinical validation will be essential to further protect the intraoperative intraocular environment and improve long-term visual outcomes.

## 1. Introduction

Cataract surgery is performed within a highly dynamic intraocular environment in which fluid exchange, pressure regulation, and tissue biomechanics interact continuously [1,2,3,4]. During phacoemulsification, subtle imbalances between inflow and outflow can rapidly destabilize the anterior chamber and propagate mechanical stress to adjacent ocular structures [2,3,4]. The eye comprises multiple interacting microenvironments, including the anterior chamber, vitreous cavity, and ocular fundus, each characterized by a delicate physical and biochemical balance that may be disrupted by surgical maneuvers [5,6,7,8]. Understanding how these intraocular microenvironments respond to surgical stressors is therefore essential for optimizing safety and functional outcomes.

Cataract surgery remains the most frequently performed ophthalmic procedure worldwide and one of the most cost-effective medical interventions [9,10,11,12,13]. The introduction of phacoemulsification has significantly improved postoperative outcomes, visual rehabilitation, and safety profiles. Nevertheless, cataract surgery is a highly dynamic intraocular intervention in which the stability of the intraocular environment, particularly the balance between inflow and outflow, is a decisive factor in surgical safety and visual outcomes [14,15,16]. In this context, intraoperative complications related to fluid management remain a relevant concern.

Among these, the post-occlusion break surge represents one of the most critical sources of anterior chamber instability and surgical risk [17,18,19,20,21]. A surge occurs when the aspiration line of a phacoemulsification system becomes occluded by lens fragments or viscoelastic material, leading to a progressive increase in vacuum [17]. Once the obstruction is relieved, the sudden equilibration between aspiration and infusion results in a rapid outflow of aqueous humor from the anterior chamber. This abrupt volumetric change disrupts the intraocular environment and may result in chamber shallowing, posterior capsule rupture, vitreous loss, or even dropped nucleus fragments. Such complications not only jeopardize surgical safety but also increase the risk of postoperative sequelae such as cystoid macular edema, retinal detachment, and long-term visual deterioration [17,19,20,22,23,24,25].

The magnitude of surge is determined by multiple interacting factors: (i) the compliance of aspiration tubing and cassettes, (ii) the amount of entrapped air in the system, (iii) the preset vacuum limits and infusion pressure, and (iv) the intrinsic compliance of the human eye, which varies with age, axial length, and ocular rigidity. Over the last two decades, experimental models, ranging from rigid chambers to collapsible artificial anterior chambers and mechanical spring-eye models, have advanced our understanding of surge dynamics and their impact on anterior chamber stability [22,23,26,27].

Technological innovation has paralleled these experimental insights. Early strategies such as low-compliance tubing and aspiration bypass systems were followed by more sophisticated solutions, including pressurized, gas-forced, and ultimately active and adaptive fluidics technologies [28,29]. More recently, the incorporation of pressure sensors directly in the handpiece, as in the Active Sentry system, has enabled real-time modulation of vacuum release and infusion compensation, redefining how the intraocular environment is dynamically regulated during surgery [30,31,32].

Despite these advances, the complete elimination of surge remains elusive. Therefore, a detailed understanding of its mechanisms, measurement methods, technological control systems, and clinical implications is essential for both researchers and cataract surgeons. From a broader perspective, a surge represents a paradigmatic example of how alterations in the intraocular microenvironment during surgery may directly translate into structural and functional complications.

Because of that, the objective of this review is to provide a comprehensive synthesis of current knowledge on post-occlusion break surge in phacoemulsification, integrating laboratory and clinical evidence, technological innovations, and strategies for prevention. By framing surge as a key disruptor of the intraocular environment during cataract surgery, this article aims to outline the state of the art and highlight future directions in fluidics safety and anterior chamber stability.

## 2. Materials and Methods

### 2.1. Review Design

This work is a narrative review based on a structured literature search. The aim was to identify experimental, mechanical, and clinical studies analyzing occlusion break surge and anterior chamber stability in phacoemulsification systems. Because of the heterogeneity of study designs, experimental setups, and outcome measures, this article is not a formal systematic review or meta-analysis. Instead, it synthesizes key concepts and trends from bench, ex vivo, and modeling studies, as well as clinical evidence, related to intraoperative fluidics and the stability of the intraocular environment during cataract surgery.

### 2.2. Databases and Search Strategy

Electronic searches were performed in PubMed, Embase, and Scopus from January 2010 to December 2025, with the last search conducted on 15 December 2025. A representative PubMed search strategy was (phacoemulsification OR cataract surgery) AND (occlusion break OR surge) AND (fluidics OR vacuum OR anterior chamber stability OR intraocular pressure OR pressure control OR pressure sensor OR active fluidics OR adaptive fluidics). Equivalent terms and controlled vocabulary were adapted for Embase and Scopus.

To complement the database search, additional references were identified by manually screening the reference lists of relevant articles and by reviewing manufacturer technical reports when they provided quantitative information on surge behavior or intraocular pressure (IOP) stability.

### 2.3. Eligibility Criteria and Study Selection

Studies were considered eligible for inclusion if they evaluated fluidics performance, occlusion break surge behavior, or anterior chamber stability in phacoemulsification systems using experimental, bench, ex vivo, cadaveric, finite element, or clinical designs. Publications describing mechanical or computational models of ocular compliance and surge dynamics were also included. The review focused on articles published between 2010 and 2025 in peer-reviewed journals, as well as selected manufacturer technical reports when these provided quantitative data on surge magnitude, aqueous volume displacement, or intraoperative IOP behavior. Seminal studies published before 2010 were also included when they were considered foundational for the understanding of post-occlusion break surge, vacuum dynamics, measurement methodologies, or the historical evolution of phacoemulsification fluidics. These earlier studies were incorporated to provide essential conceptual and methodological context for the more recent evidence.

Studies were excluded if they addressed surgical procedures unrelated to phacoemulsification, consisted solely of single case reports without quantitative fluidics measurements, or lacked sufficient methodological detail to allow meaningful interpretation of surge-related outcomes. The initial database search retrieved 1142 records. After removal of duplicates, 836 unique records remained and were screened at the title and abstract level. Of these, 214 articles were selected for full-text review based on relevance to intraoperative fluidics and anterior chamber stability. Following full-text assessment, 54 peer-reviewed publications and two manufacturer technical documents met the inclusion criteria and were incorporated into the narrative synthesis. Given the narrative nature of this review and the heterogeneity of experimental models, platforms, and outcome measures, no formal risk-of-bias assessment or pooled quantitative synthesis was performed. Instead, the included evidence was organized into major thematic domains reflecting the pathophysiology of surge, experimental measurement methods, technological evolution of fluidics systems, comparative platform performance, clinical implications, and current and emerging strategies for stabilizing the intraocular environment during cataract surgery.

### 2.4. Overview of Study Categories Included in the Narrative Synthesis

Table 1 summarizes the main types of studies and models included in this narrative synthesis, highlighting representative references, experimental or clinical settings, and key outcomes related to surge magnitude and anterior chamber stability.

## 3. Results

### 3.1. Pathophysiology of Post-Occlusion Break Surge

#### 3.1.1. Basic Mechanisms

During phacoemulsification, occlusion occurs when the aspiration port of the handpiece becomes blocked by nuclear or cortical lens fragments, iris tissue, or ophthalmic viscosurgical devices [17,19,20]. While the port is occluded, the peristaltic, Venturi, or diaphragm pump continues to aspirate, leading to a progressive rise in vacuum pressure within the aspiration line and cassette [17,19]. During this phase, mechanical energy is stored in the elastic deformation of the tubing and cassette walls, as well as in the compression of entrapped air bubbles within the system [19].

When the occlusion suddenly breaks, this stored energy is released almost instantaneously. The aspiration tubing recoils, entrapped air rapidly decompresses, and a large volume of fluid is abruptly drawn from the anterior chamber into the aspiration system. Because the irrigation inflow cannot immediately compensate for this sudden demand, a sharp volumetric deficit develops, resulting in abrupt shallowing or even transient collapse of the anterior chamber [19,20]. This sudden disruption represents the fundamental biomechanical event underlying post-occlusion break surge and the associated instability of the intraocular environment.

#### 3.1.2. Determinants of Surge Magnitude

The magnitude of the post-occlusion break surge is governed by multiple interrelated factors. One of the most important aspects is the compliance of the aspiration system: flexible tubing and compliant cassettes store greater elastic potential energy during vacuum buildup, thereby amplifying the surge at the moment of occlusion release. The presence of entrapped air further exaggerates this effect, as microbubbles act as elastic reservoirs that markedly amplify volume shifts upon decompression.

The preset vacuum limit is another critical determinant. Higher vacuum settings increase the amount of stored potential energy and, consequently, the surge amplitude. Laboratory studies consistently demonstrate a proportional relationship between vacuum limit and surge volume across different phacoemulsification platforms.

Infusion pressure also plays a central role. Conventional gravity-based fluidics rely on bottle height to generate irrigation pressure; however, this passive system cannot respond rapidly enough to compensate for the sudden outflow during a surge, leading to transient chamber instability. Finally, the intrinsic biomechanical compliance of the human eye strongly modulates the IOP–volume relationship. Ocular compliance is nonlinear and age-dependent: younger eyes and highly myopic eyes tend to be more compliant and are therefore particularly vulnerable to exaggerated chamber shallowing during surge events [20,28,30,35].

#### 3.1.3. Consequences on Intraocular Structures

The volumetric loss associated with a surge has been reported to range from approximately 7% to 66% of the anterior chamber volume (ACV) in a phakic adult eye, assuming a reference ACV of approximately 200–300 µL. This value varies wiith axial length and anterior segment anatomy, and is influenced by the phaco platform, vacuum settings, and fluidics configuration [20,21,36]. Such abrupt shifts in intraocular volume and pressure can have multiple structural consequences.

Anterior chamber flattening significantly increases the risk of posterior capsule rupture and vitreous prolapse. Rapid IOP fluctuations may reduce the corneal endothelium’s safety margin and transiently impair ocular perfusion. In addition, posterior displacement of the capsule and sudden vitreoretinal traction may occur, potentially promoting posterior vitreous detachment and, in susceptible eyes, more severe retinal complications [20,23]. These effects highlight that surge is not only a fluidics disturbance but a biomechanical stressor affecting multiple intraocular tissues.

#### 3.1.4. Clinical Relevance

Post-occlusion break surge is therefore not merely a laboratory phenomenon but a clinically significant intraoperative event. By directly destabilizing the anterior chamber and inducing abrupt IOP fluctuations, surge represents a major threat to intraoperative safety and has important downstream implications for postoperative recovery and visual outcomes. For these reasons, understanding the pathophysiology of surge and developing effective strategies for its mitigation remain central challenges in the design of modern phacoemulsification fluidics systems and in contemporary cataract surgery.

### 3.2. Measurement Methods for Surge

#### 3.2.1. Historical Approaches: Pressure-Based Models

The earliest investigations of post-occlusion break surge relied on rigid artificial anterior chambers equipped with pressure sensors. In these experimental setups, a sudden drop in IOP immediately following occlusion release was used as a surrogate marker of surge intensity [17,19]. Although these models were highly reproducible and technically simple, they failed to replicate the biomechanical compliance of the human eye. As a result, they often exaggerated negative pressure peaks and underestimated the true magnitude of volumetric shifts occurring during surge events [37].

#### 3.2.2. Collapsible Anterior Chamber Models

To overcome the limitations of rigid systems, collapsible artificial anterior chamber models were developed. These devices more accurately reproduce the physiological behavior of the anterior chamber walls, in which a pressure drop results in a measurable reduction in chamber volume rather than extreme negative pressures. Using these models, surge characterization showed smaller pressure changes but more realistic volume fluctuations than in rigid chambers [31,37,38]. This approach represented an important step toward reproducing the dynamic behavior of the intraocular environment during phacoemulsification.

#### 3.2.3. Spring-Eye Model

The introduction of the so-called “spring-eye” model marked a major advance in surge measurement [22]. This system incorporates a piston supported by a series of calibrated springs designed to emulate human ocular compliance. Laser displacement sensors quantify piston movement in real time, allowing direct calculation of volumetric displacement during occlusion break events. This model enabled robust inter-system comparisons under standardized conditions and served as a reference platform for the experimental evaluation of phacoemulsification fluidics [20,29,31,36,38].

#### 3.2.4. Mechanical Compliance Models

Building on cadaveric biomechanical data, Dyk and Miller developed a mechanical compliance model based on exponential pressure–volume relationships derived from enucleated human eyes. The system integrates a displaceable piston with serially engaged springs, generating volumetric responses that closely match those observed in human ocular tissue [22]. This model allows highly standardized, reproducible, and physiologically relevant quantification of surge behavior across different phaco platforms and settings.

#### 3.2.5. Ex Vivo and Cadaveric Eye Studies

Direct testing on donor or cadaveric eyes provides the most anatomically realistic simulation of human ocular structures and tissue properties. These studies allow direct observation of anterior chamber behavior, capsular movement, and vitreoretinal responses during surge. However, their broader application is limited by inter-specimen variability, restricted tissue availability, and progressive mechanical fatigue with repeated testing [21,22,23,31,37].

#### 3.2.6. Proposed Indicators for Surge Characterization

Across the different experimental and ex vivo methodologies, several quantitative parameters have been proposed to characterize surge performance in a standardized manner [14,15,18,22,29,31]. These include the peak volume fluctuation (expressed in microliters or as a percentage of total chamber volume), the half recovery time, defined as the time required for the anterior chamber to return to baseline after the surge, the surge area (µL·s), which integrates both magnitude and duration, and the recovery slope, reflecting the rate of pressure or volume restoration after occlusion break. Together, these metrics provide a more comprehensive and physiologically meaningful assessment of surge behavior than pressure-only measurements and enable more reliable comparisons between phacoemulsification systems.

The main experimental and ex vivo approaches currently used for surge quantification, together with their respective advantages, limitations, and measured parameters, are summarized in Table 2.

### 3.3. Evolution of Fluidics Systems

#### 3.3.1. Gravity Fluidics

Gravity-driven irrigation was the standard method for maintaining anterior chamber stability during phacoemulsification for several decades. Balanced salt solution (BSS) bottles were elevated above the patient’s eye to generate hydrostatic pressure and sustain inflow [17]. Although simple and robust, this approach was inherently limited. Irrigation pressure was relatively fixed and could not respond dynamically to real-time fluctuations in aspiration flow. As a result, IOP and anterior chamber depth frequently fluctuated, particularly during occlusion break events. Surge mitigation depended almost exclusively on manual bottle height adjustments performed by the surgeon, a delayed and imprecise intervention that offered only partial protection against chamber instability [14,15,19,20,21,40,41].

#### 3.3.2. Hyper-Pressurized and Gas-Forced Infusion

To overcome the intrinsic limitations of gravity-based systems, hyper-pressurized infusion technologies were subsequently developed. By increasing infusion pressure above that achievable through gravity alone, these systems aimed to counteract the sudden outflow associated with surge [14]. While this strategy reduced the incidence and severity of chamber collapse, it did not fully eliminate surge-related instability [14,15,18,19].

Gas-forced infusion represented a further step forward by actively pressurizing the irrigation line with compressed gas [25]. Experimental studies demonstrated that this approach could effectively prevent anterior chamber collapse and minimize endothelial cell loss by rapidly compensating for the volume loss caused by aspiration. However, this benefit came at the cost of operating at consistently high intraocular pressures, raising safety concerns about ocular perfusion and potential pressure-induced tissue stress [17,24,31].

#### 3.3.3. Active Fluidics

fA major paradigm shift occurred in 2013 with the introduction of Active Fluidics in the Centurion Vision System (Alcon; Fort Worth, TX, USA) [33]. Instead of relying on static bottle height, this technology employs a BSS bag housed within the surgical console and compressed by mechanical plates under the control of integrated pressure sensors. Surgeons can set a target IOP, and the system dynamically adjusts infusion pressure in real time to maintain this level throughout the procedure [29,32,41,42,43,44,45].

Active Fluidics significantly improved anterior chamber stability and enabled phacoemulsification at lower, more physiologic IOPs [32]. This translated into reduced corneal stress, improved endothelial protection, and enhanced patient comfort during surgery [29,32,41,42,43]. The concept of maintaining a controlled intraoperative intraocular environment rather than merely counteracting surge marked a fundamental evolution in fluidics design.

#### 3.3.4. Adaptive Fluidics

Adaptive Fluidics, introduced by Bausch & Lomb with the Stellaris Elite platform (Bausch & Lomb, Bridgewater, NJ, USA) [46], applies a different control strategy. Instead of compressing a fluid bag, the system modulates the air pressure in the infusion bottle in direct response to changes in vacuum. This allows real-time irrigation compensation synchronized with aspiration demand.

By proactively adjusting inflow rather than reacting to volume loss, Adaptive Fluidics provides measurable improvements in anterior chamber stability compared with traditional gravity-based systems. These benefits are particularly evident in challenging clinical scenarios, such as highly myopic eyes that are prone to lens–iris diaphragm retropulsion syndrome [30,32,44].

#### 3.3.5. Handpiece-Integrated Pressure Sensors (Active Sentry)

The most recent step in fluidics evolution is the integration of pressure sensors directly into the phacoemulsification handpiece, as exemplified by the Active Sentry system. Unlike conventional platforms, where pressure changes must propagate through the aspiration line to reach a cassette-based sensor, Active Sentry detects fluctuations at the tip level, virtually eliminating signal latency [33,35,40,41].

When an impending occlusion break is detected, the system partially vents vacuum before a large volume of fluid is drawn from the eye, thereby attenuating both the magnitude and duration of the surge. Experimental data consistently demonstrate that the Centurion platform equipped with Active Sentry produces lower aqueous volume losses than comparable systems, even at high-vacuum limits [31]. This innovation represents a critical step toward real-time, predictive control of the intraocular environment during cataract surgery.

### 3.4. Comparative Evidence Between Phacoemulsification Systems

#### 3.4.1. Historical Systems

Early phacoemulsification platforms, including the Legacy (Alcon, Fort Worth, TX, USA), Millennium (Bausch & Lomb, Bridgewater, NJ, USA), and Sovereign (AMO, Santa Ana, CA, USA), relied exclusively on gravity-based infusion [47]. These systems were characterized by high surge susceptibility due to the compliance of the aspiration tubing and the absence of dynamic infusion control.

To mitigate these limitations, two major technical innovations were introduced. The Aspiration Bypass System (ABS) allowed a small amount of inflow during occlusion, partially venting trapped vacuum, while Cruise Control (CC) functioned as a flow restrictor that smoothed occlusion breaks [48,49,50]. Despite these improvements, significant chamber instability persisted at vacuum levels above 400 mmHg [17,24]. Comparative studies demonstrated that the Infiniti platform (Alcon, Fort Worth, TX, USA), which incorporated ABS and lower-compliance tubing, achieved approximately 18% lower surge than the Legacy system when operating at 500 mmHg [51].

For cross-platform comparability, surge volumes expressed as percentages were normalized to a reference anterior chamber volume (ACV) of approximately 250 µL for a phakic adult eye, consistent with prior experimental and modeling studies. Actual ACV varies with axial length, anterior segment anatomy, and age; therefore, percentage values should be interpreted as normalized estimates rather than patient-specific measurements.

#### 3.4.2. Intermediate Platforms

Between 2005 and 2015, the Whitestar Signature Pro (Johnson & Johnson Vision, Jacksonville, FL, USA) [52] and the Stellaris PC (Bausch & Lomb, Bridgewater, NJ, USA) [46] represented a transitional generation toward more versatile fluidics systems.

In Whitestar Signature Pro platforms, reported surge volumes ranged from 30 to 103 µL, corresponding to 12–41% of anterior chamber volume [42,52]. In contrast, Stellaris PC exhibited higher surge values, ranging from 67 to 163 µL (27–65%), with greater inter-experimental variability [46,47]. Both systems allowed operation in either peristaltic or Venturi modes; however, Venturi-driven aspiration consistently produced larger surge magnitudes. Overall, these platforms achieved improved chamber stability compared with historical gravity-based systems, but performance remained unreliable at high vacuum levels [47].

#### 3.4.3. Modern Platforms

The introduction of the Centurion Vision System (Alcon; Fort Worth, TX, USA) marked a major step forward by incorporating Active Fluidics, based on bag compression and sensor-driven, real-time pressure feedback [33]. Using this technology, reported surge volumes ranged from 17 to 77 µL, corresponding to 7–31% of the anterior chamber volume [29,32].

With the addition of the Active Sentry handpiece sensor, which detects occlusion breaks directly at the tip and vents vacuum preemptively, surge performance was further improved [40,53,54]. Reported surge volumes were ≤74.7 µL, with aqueous losses consistently below 30% [53,54].

In contrast, the EVA platform (DORC, Zuidland, The Netherlands), characterized by higher system compliance, showed larger surge volumes of 47–165 µL, corresponding to aqueous losses of up to 66% [20,55]. The Legion platform (Alcon) exhibited surge volumes of around 70 µL, comparable to those of the Signature Pro system, but with improved ergonomics and workflow integration [44,47].

#### 3.4.4. Quatera 700 (Zeiss)

A separate analysis is warranted for the Quatera 700 (Carl Zeiss Meditec AG, Jena, Germany), introduced in 2021 and based on Centrally Controlled Fluidics (CCF). In this architecture, infusion and aspiration are electronically synchronized in real time, independently of bottle height or bag compression mechanisms [56,57].

Laboratory evaluations have reported surge durations of 239–471 ms, consistently shorter than those observed with the Centurion system (Alcon; Fort Worth, TX, USA) under matched vacuum and flow conditions [31,56]. Peak surge volumes in spring-eye models were slightly higher than those observed with Centurion at equivalent settings, but these were compensated by rapid pressure recovery [56]. This combination suggests superior temporal control of IOP, thereby reducing the clinical impact of surge despite similar volumetric excursions [42,44,47,54].

At present, a limitation of Quatera 700 (Carl Zeiss Meditec AG, Jena, Germany) remains the relative scarcity of large, independent clinical datasets compared with more established platforms such as Centurion [56].

#### 3.4.5. Unity VCS (Next-Generation Predictive Infusion)

The most recent evolution in fluidics technology is represented by the Unity Vision Control System (Unity VCS; (Alcon; Fort Worth, TX, USA)), which introduces a predictive infusion paradigm. Instead of reacting to vacuum rise, predictive algorithms adjust irrigation pressure before maximum vacuum is reached, thereby minimizing surge at the moment of occlusion break [33].

Preliminary data indicate aqueous volume losses consistently below 60 µL, corresponding to less than 20% of anterior chamber volume. Surge recovery times are estimated to lie in the 200–300 ms range, although full peer-reviewed validation is still pending [33]. An additional advantage of this system is its ability to operate at physiologic IOPs (20–40 mmHg), thereby reducing endothelial stress [25]. However, large-scale, independent clinical validation remains necessary.

#### 3.4.6. Comparative Perspective

From a comparative standpoint, the Centurion platform equipped with Active Sentry (Alcon; Fort Worth, TX, USA) currently represents the most extensively validated system, combining low surge volume with rapid recovery [56]. The Quatera 700 (Carl Zeiss Meditec AG, Jena, Germany) demonstrates shorter surge duration but slightly higher peak volumes, balanced by tight IOP control and rapid pressure restitution [56]. The Unity VCS (Alcon; Fort Worth, TX, USA) shows early, predominantly manufacturer-reported and bench-level evidence of the lowest aqueous volume losses, achieved through predictive control and consistent physiologic IOP targets; however, direct, independent, like-for-like comparisons under matched experimental conditions remain limited [33]. In contrast, platforms such as EVA (DORC, Zuidland, The Netherlands) and Stellaris PC (Bausch & Lomb, Bridgewater, NJ, USA). exhibit higher surge values and therefore less reliable anterior chamber stability under demanding surgical conditions [20,46,47,55].

Together, these data illustrate the progressive refinement of fluidics systems toward proactive, sensor-driven, and predictive control of the intraoperative intraocular environment. The comparative performance of contemporary phacoemulsification systems regarding surge magnitude and recovery time is summarized in Table 3. Surge duration is reported heterogeneously across the literature. Where quantitative temporal metrics (ms) were available from bench or experimental models, these values are presented. For several platforms, however, duration is described only qualitatively (e.g., “shorter,” “variable,” or “rapid recovery”), and these descriptors are explicitly indicated as such in Table 3. Where performance claims are derived from manufacturer technical documentation rather than peer-reviewed sources, this is explicitly stated, and such data should be interpreted as preliminary and hypothesis-generating.

#### 3.4.7. Dedicated Analysis: Quatera 700 and Unity VCS

Among the most recent phacoemulsification platforms, the Quatera 700 (Carl Zeiss Meditec AG, Jena, Germany) [34] and the Unity Vision Control System (Unity VCS, (Alcon; Fort Worth, TX, USA)) [33] represent significant advances in fluidics technology. Both systems were specifically designed to minimize post-occlusion break surge while enabling surgery to be performed at physiologic IOP levels, thereby improving control of the intraoperative intraocular environment [15,16].

The Quatera 700, introduced in 2021, is based on a novel Centrally Controlled Fluidics (CCF) architecture [34]. Unlike conventional systems that rely on gravity-driven infusion or bag compression, Quatera electronically synchronizes infusion and aspiration in real time through an integrated, centrally regulated fluidics module.

Laboratory evaluations have reported surge durations of 239–471 ms, with rapid pressure recovery and minimal chamber instability, even under high-vacuum conditions [31]. The system maintains a constant anterior chamber depth and provides precise pressure restitution following occlusion breaks, ensuring stable anterior segment conditions throughout surgery [34,44,56].

From a clinical perspective, by maintaining a tightly regulated fluidics environment, the Quatera 700 may reduce the risk of intraoperative complications such as posterior capsule rupture and lens–iris diaphragm retropulsion syndrome. However, despite promising experimental performance, the availability of large, long-term independent clinical datasets remains more limited than for more established platforms.

The Unity Vision Control System (Alcon; Fort Worth, TX, USA) represents the newest generation of predictive fluidics. Rather than reacting to vacuum rise, Unity VCS uses real-time predictive algorithms to adjust irrigation pressure before peak vacuum is reached, thereby proactively minimizing surge at the moment of occlusion break [33].

Preliminary bench data and manufacturer-reported technical information suggest aqueous volume losses of <60 µL, corresponding to <20% of anterior chamber volume, with surge recovery times of approximately 200–300 ms under specific experimental settings [31]. In addition to predictive pressure modulation, Unity VCS incorporates optimized tubing and cassette designs with reduced compliance, which further limits surge magnitude.

A proposed advantage of this platform is its ability to operate at low, physiologic IOP levels (20–40 mmHg), as described in manufacturer technical documentation [33], aligning with the current trend toward safer, less invasive cataract surgery. However, large-scale, independent clinical validation is still required to confirm its long-term efficacy and safety.

Both the Quatera 700 and Unity VCS (Alcon; Fort Worth, TX, USA) represent a departure from traditional gravity-based and bag-compression fluidics systems. The Quatera 700 emphasizes tight synchronization of infusion and aspiration with rapid pressure recovery, whereas Unity VCS focuses on predictive algorithms and sustained operation at physiologic IOP. When compared with the Centurion system equipped with Active Sentry (Alcon; Fort Worth, TX, USA), which currently represents the most extensively validated reference platform, both systems demonstrate competitive surge performance, with Unity VCS approaching even lower aqueous-volume-loss thresholds.

Together, these platforms illustrate the future trajectory of phacoemulsification fluidics, in which the objective extends beyond simple surge suppression toward the active, continuous maintenance of a stable and physiologic intraocular environment across all surgical scenarios.

## 4. Discussion

### 4.1. Clinical Implications of Post-Occlusion Break Surge

#### 4.1.1. Intraoperative Safety

The most immediate consequence of a surge is the abrupt shallowing of the anterior chamber [17,19,20]. When the posterior capsule lies close to the phaco tip at the moment of occlusion release, the sudden fluid outflow can precipitate posterior capsule rupture, vitreous prolapse, and even dropped nuclear fragments [19,22,37,38,39,43]. These complications not only prolong surgical time but also significantly increase the risk of endophthalmitis, retinal detachment, and poor visual outcomes [19,22].

In addition to capsular complications, the surge destabilizes multiple intraocular structures. Rapid fluctuations in anterior chamber depth may induce iris billowing, phacodonesis, and disruption of the lens–iris diaphragm. In extreme cases, particularly in highly myopic eyes, surge has been implicated in lens–iris diaphragm retropulsion syndrome, a condition associated with sudden chamber deepening, iris concavity, and marked intraoperative instability [22].

#### 4.1.2. Corneal and Endothelial Impact

Anterior chamber collapse during surge markedly increases the likelihood of corneal endothelial injury, especially when the phaco tip or nuclear fragments approach the posterior corneal surface [29]. Moreover, repeated intraoperative IOP fluctuations impair corneal perfusion and may contribute to postoperative corneal edema [25,29].

Clinical trials comparing active fluidics systems with traditional gravity-based infusion have consistently demonstrated reduced endothelial compromise when anterior chamber stability is maintained [41,45,47,55]. These findings emphasize that surge control is not only a matter of chamber stability but also a critical determinant of corneal tissue preservation.

#### 4.1.3. Posterior Segment Implications

Although surge has traditionally been regarded as an anterior segment phenomenon, increasing evidence indicates that it can also affect the posterior segment. Sudden anterior chamber volume loss generates acute posterior vitreous traction, which has been associated with posterior vitreous detachment (PVD) [29,61]. If PVD occurs prematurely, it may predispose to retinal tears or rhegmatogenous retinal detachment, particularly in eyes with high myopia or preexisting vitreoretinal adhesions [29].

In addition, repeated intraoperative IOP fluctuations may compromise the blood–retinal barrier, thereby increasing the risk of cystoid macular edema. Geng et al. demonstrated that eyes exposed to greater anterior chamber instability showed significantly higher rates of PVD and postoperative macular thickening than eyes stabilized with anterior chamber maintainers.

#### 4.1.4. Patient Outcomes and Long-Term Vision

The downstream consequences of intraoperative surge may include delayed visual recovery, prolonged corneal edema, and an increased risk of sight-threatening complications in both the anterior and posterior segments [62]. Therefore, maintaining anterior chamber stability is not merely a technical objective but a critical determinant of patient safety and postoperative visual quality.

From a broader perspective, the clinical impact of surge underscores the importance of maintaining a stable, physiologic intraocular environment throughout phacoemulsification, as even brief episodes of instability can have lasting visual consequences.

### 4.2. Current Strategies to Minimize Surge

#### 4.2.1. Optimization of Fluidics Parameters

One of the most straightforward strategies to reduce surge risk is adjusting vacuum and aspiration flow limits [17,24]. Lower vacuum settings and reduced aspiration rates decrease the elastic potential energy stored within the aspiration tubing, thereby limiting surge magnitude. However, this mechanical advantage comes at the expense of slower nuclear fragment aspiration and reduced surgical efficiency.

A balanced approach is therefore required, in which fluidics parameters are tailored to nuclear density, ocular anatomy, surgeon experience, and surgical technique to optimize both safety and efficiency while preserving the stability of the intraocular environment.

#### 4.2.2. Vacuum Management

An appropriate balance of the selected vacuum level is central to surge mitigation. Higher vacuum improves nuclear fragment holding and followability, but it also increases the amount of mechanical energy that accumulates in the aspiration pathway during occlusion. When the occlusion breaks, this stored energy is released abruptly and can translate into a larger transient outflow and a greater risk of anterior chamber shallowing, particularly under high-flow conditions or in eyes with lower ocular rigidity [14,17,24,57].

From a systems perspective, the aspiration line, cassette, and any entrapped air behave as a compliant hydraulic circuit. The greater the compliance of the tubing and cassette, the larger the volume excursion that can occur for a given pressure change. As a result, surge risk is influenced not only by the vacuum limit selected on the console, but also by platform-specific design features that modify system compliance (e.g., low-compliance tubing, cassette architecture, and venting strategies), as well as by patient-specific factors that determine ocular compliance [19,20,22,30,31].

Importantly, not all measured vacuum reflects true occlusion at the phaco tip. Even in the absence of port blockage, aspiration flow through the tubing generates a baseline resistive pressure drop, often referred to as a passive vacuum component. This resistive component depends on the aspiration flow rate and the hydraulic resistance of the aspiration pathway and can influence how rapidly the system approaches the preset vacuum limit and how the console interprets vacuum rise during dynamic surgical maneuvers [17,19,24,30,31].

Clinically, vacuum management should be approached as a trade-off between safety and efficiency. Lowering the vacuum limit can reduce surge magnitude and anterior chamber instability, but excessive reductions may compromise fragment followability, prolong nuclear removal, and increase overall surgical manipulation. Practical surge mitigation therefore relies on balancing vacuum and aspiration flow with an appropriate infusion strategy and a realistic IOP target, tailored to cataract density, incision stability, anterior segment anatomy, and the specific fluidics architecture of the chosen platform [24,30,31].

Modern phacoemulsification systems continuously monitor pressure within the aspiration line and compare it with the surgeon-defined vacuum limit. When the measured pressure approaches or exceeds this threshold, the pump can decelerate, pause, or initiate venting mechanisms to limit further vacuum buildup and facilitate controlled pressure recovery. Although these automated responses improve chamber stability, the surgeon’s selection of vacuum and flow parameters remains a primary and adjustable determinant of both surge risk and surgical efficiency across a wide range of intraoperative scenarios [17,24,57].

#### 4.2.3. Infusion Pressure Management

Infusion pressure remains a central determinant of anterior chamber stability during phacoemulsification [32]. In gravity-driven systems, irrigation pressure is controlled exclusively by bottle height [14]. Although increasing bottle height may reduce surge magnitude, it often results in excessive IOP, which can compromise ocular perfusion and distort the normal anatomy of intraocular structures. Sustained elevations in IOP may also alter the physiologic conditions of the intraocular microenvironment, including corneal endothelial function, retinal and optic nerve head perfusion, and the biomechanical behavior of the vitreous body, particularly in vulnerable eyes such as those with advanced glaucoma or high myopia [14,40].

In contrast, active fluidics platforms dynamically regulate irrigation pressure in real time, allowing surgeons to maintain target IOPs much closer to physiologic levels (20–40 mmHg) while still preventing chamber collapse [32,40,41,43,47,61]. This control paradigm shifts infusion management from a static, height-based system to a closed-loop, pressure-targeted strategy, improving the balance between mechanical stability and biologic safety across a wider range of surgical conditions and patient anatomies.

#### 4.2.4. Advanced Fluidics Platforms

Several modern platforms now incorporate automated mechanisms specifically designed to minimize surge and stabilize the intraoperative intraocular environment. Active Fluidics (Centurion; (Alcon; Fort Worth, TX, USA)) automatically adjusts the compression of balanced salt solution bags to maintain IOP at the surgeon-defined target, resulting in consistently lower surge volumes across a wide range of vacuum settings [29]. Adaptive Fluidics (Stellaris Elite AFS; (Bausch & Lomb, Bridgewater, NJ, USA)) modulates air pressure within the infusion bottle in response to changing vacuum demand, improving chamber stability, particularly in highly myopic eyes [44,46].

The Active Sentry handpiece (Centurion; (Alcon; Fort Worth, TX, USA)) integrates a pressure sensor directly at the phaco tip, enabling earlier detection of rapid pressure transients and automated vacuum venting at the moment of occlusion release. Bench and experimental studies suggest that this architecture can reduce both the magnitude and the duration of surge events compared with conventional, cassette-level sensing, particularly under high-flow or high-vacuum conditions [56].

More recently, next-generation systems such as Quatera 700 and Unity VCS have introduced predictive or centrally controlled fluidics concepts aimed at maintaining a predefined IOP setpoint while dynamically modulating infusion and aspiration. Early evidence from bench models and manufacturer technical documentation indicates low fluid loss and rapid pressure recovery; however, independent, peer-reviewed clinical validation across matched surgical settings remains limited, and comparative performance should therefore be interpreted cautiously [33,44,56].

#### 4.2.5. Auxiliary Devices and Surgical Techniques

In addition to platform-based solutions, several auxiliary devices and surgical techniques can further enhance chamber stability. Aspiration Bypass System tips and cruise control mechanisms allow partial venting of trapped vacuum during occlusion, thereby reducing the abruptness of occlusion break events [28,29].

Anterior chamber maintainers provide continuous inflow and are particularly useful during instrument exchange, in eyes with large incisions, or in the presence of zonular weakness. Ophthalmic viscosurgical devices (OVDs) can temporarily restore chamber depth after a surge event and provide a protective cushion for the corneal endothelium [17].

Proper incision architecture is also critical, as tight, self-sealing wounds minimize fluid leakage and reduce instability during aspiration [63]. In addition, appropriate sleeve and tip selection, particularly narrow-bore tips with optimized sleeve fit, can limit abrupt aspiration flow surges while preserving efficient fluidics [64,65].

#### 4.2.6. Intraoperative Maneuvers

Surge prevention also depends on surgeon behavior and intraoperative strategy. Anticipating occlusion can be achieved through careful modulation of footswitch control and by briefly pausing ultrasound and aspiration at the moment of release, a maneuver commonly referred to as the “vent, stop, and re-form” technique, which can significantly reduce the severity of chamber instability [66,67]. Surgeons should also maintain immediate availability of OVDs for rapid chamber reformation when required.

Continuous monitoring of real-time fluidics feedback from the surgical console allows dynamic adjustment of IOP targets, vacuum limits, and aspiration flow in response to intraoperative events, further improving control of the intraocular environment [54,68,69].

#### 4.2.7. Integrated Prevention Approach

Ultimately, no single intervention completely eliminates post-occlusion break surge. Effective prevention, therefore, requires a multimodal, integrated strategy that combines appropriate platform selection (favoring active or adaptive fluidics when available), careful adjustment of vacuum, aspiration flow, and IOP targets, judicious use of auxiliary devices and OVDs, and continuous surgeon vigilance with proactive intraoperative maneuvers.

Together, these complementary measures minimize both the incidence and severity of surge, thereby reducing intraoperative complications and protecting the structural and functional integrity of both the anterior and posterior segments of the eye.

### 4.3. Future Directions in Surge Prevention and Fluidics Optimization

#### 4.3.1. Surgery at Physiologic Intraocular Pressures

A growing paradigm in modern cataract surgery is the transition toward operating at physiologic IOP levels during phacoemulsification. Traditional fluidics systems often required infusion pressures of 60–80 mmHg to counteract high-vacuum aspiration, thereby increasing corneal endothelial stress and compromising ocular perfusion [32,39,42].

Contemporary active fluidics platforms now enable stable anterior chamber maintenance at lower target IOPs (20–40 mmHg), which may reduce endothelial cell loss, improve postoperative corneal clarity, and enhance patient comfort. Future fluidics technologies are expected to further refine this approach, combining high surgical efficiency with minimal physiologic disruption of the intraocular environment.

#### 4.3.2. Integration of Intraoperative Imaging

Intraoperative swept-source optical coherence tomography has emerged as a valuable research tool for the dynamic assessment of anterior chamber depth, capsular bag fluctuations, and vitreoretinal traction during phacoemulsification [70,71,72]. Although not yet routinely integrated into commercial surgical platforms, OCT incorporation into phaco consoles could enable real-time detection of surge events and trigger automated adjustments of infusion and aspiration parameters.

Such real-time imaging feedback may also help prevent lens–iris diaphragm retropulsion syndrome and posterior vitreous detachment by identifying abnormal dynamic deformations of intraocular structures before irreversible stress is applied.

#### 4.3.3. Improved Models of Ocular Compliance

Despite important advances in experimental methods, such as collapsible artificial chambers and mechanical spring-eye models, current systems still fail to fully replicate the biological variability of human ocular compliance across different ages, refractive errors, and pathological conditions.

Future research should focus on developing biomechanically accurate ocular models that integrate regional scleral stiffness, corneal viscoelasticity, and vitreoretinal adhesion mechanics. Such models would enable more precise preclinical testing, improved inter-platform comparisons, and, potentially, individualized intraoperative risk prediction.

#### 4.3.4. Artificial Intelligence and Predictive Fluidics

The next frontier in surge prevention lies in AI-driven predictive fluidics. By continuously analyzing intraoperative datasets, such as vacuum rise profiles, aspiration flow rates, and real-time compliance responses, machine learning algorithms may predict an imminent occlusion break and preemptively modulate infusion pressure or vent vacuum before a surge fully develops.

Early implementations of predictive irrigation already exist in platforms such as Unity VCS (Alcon; Fort Worth, TX, USA), but future systems are expected to incorporate deep-learning models trained on thousands of surgical cases, enabling adaptive, patient-specific surge prevention and further stabilization of the intraoperative intraocular environment.

#### 4.3.5. Toward Comprehensive Intraocular Safety Platforms

Ultimately, fluidics optimization represents only one component of a broader intraoperative safety ecosystem. Future phacoemulsification platforms are expected to integrate multiple layers of protection, including

(i) endothelial protection modules capable of adjusting parameters when corneal stress is detected;
(ii) posterior segment monitoring, providing early warnings of abnormal vitreoretinal traction; and
(iii) automated device coordination, synchronizing phaco tip power, irrigation pressure, and aspiration rates in real time.

Together, these innovations would transform cataract surgery from a primarily reactive process, in which the surgeon manages complications as they occur, into a proactive, predictive system that continuously anticipates and prevents surge-related risks.

## 5. Conclusions

Post-occlusion break surge remains one of the most significant unresolved safety challenges in modern phacoemulsification. Although its key determinants, vacuum buildup, system compliance, and ocular biomechanics, are now well characterized, no currently available technology has succeeded in fully eliminating this phenomenon. Experimental evidence clearly demonstrates that both the magnitude and duration of surge vary substantially across platforms, reflecting fundamental differences in fluidics architecture and control strategies. Among contemporary systems, the Centurion equipped with Active Sentry and the Quatera 700 are supported by peer-reviewed experimental and clinical evidence, whereas the Unity Vision Control System represents an emerging, predictive platform for surge mitigation and intraoperative pressure control that is currently supported primarily by preliminary bench data and manufacturer technical reports, with independent clinical validation still in progress.

Surge must not be regarded as a benign or merely technical event. It directly destabilizes the anterior chamber, increases the risk of posterior capsule rupture and endothelial damage, and may generate secondary stress on the posterior segment, predisposing to posterior vitreous detachment, retinal detachment, and cystoid macular edema. Effective prevention, therefore, requires a multifaceted approach that combines optimal parameter selection, advanced fluidics technologies, adjunctive safety devices, and precise surgical technique.

Looking forward, further advances in cataract surgery will likely be driven by the refinement of physiologic fluidics, enabling stable surgery at intraocular pressures closer to those found in the natural eye. The integration of real-time intraoperative imaging and artificial intelligence-based predictive fluidics will likely allow earlier detection of instability and proactive surge prevention. In parallel, the development of more sophisticated biomechanical models of ocular compliance and fully integrated safety platforms will be critical to protecting both anterior and posterior segment structures.

In summary, although substantial progress has been achieved in understanding and mitigating post-occlusion break surge, its complete elimination remains an unmet need in cataract surgery. Continued technological innovation, standardized measurement methodologies, and robust independent clinical validation will be essential to ensure long-term patient safety, surgical efficiency, and visual outcomes.

## Figures and Tables

**Table 1:** Overview of study categories included in this narrative review.

Category	Sample/Model	Representative References	Study Type	Key Outcomes
Mechanical modeling	Artificial chambers	Thorne et al. [19]; Dyk et al. [22]	Experimental	ΔVmax, recovery time
Fluidics comparison	Various phaco systems	Aravena et al. [20]; Fanney et al. [31]	Bench/Cadaveric	Surge volume, duration
Active/adaptive systems	Centurion (Alcon; Fort Worth, TX, USA), Stellaris (Bausch & Lomb, Bridgewater, NJ, USA)	Luo et al. [28]; Wang et al. [32]	Clinical/lab	IOP stability
Predictive fluidics	Unity VCS (Alcon; Fort Worth, TX, USA), Quatera 700 (Carl Zeiss Meditec AG, Jena, Germany)	Alcon [33] and Zeiss [34] technical information,	Prototype	Predictive IOP control
Posterior biomechanics	Human eye models	Creveling et al. [23]	Finite element	Pressure–traction transfer

**Table 2:** Methods for quantification of post-occlusion break surge.

Method	Main Advantages	Main Limitations	Measured Parameters
Rigid chamber (non-collapsible) [37,38]	High reproducibility; simple experimental setup	Does not reproduce real ocular compliance; overestimates negative pressures	Pressure drop; recovery time
Collapsible artificial chamber [17,31,38]	More realistic simulation of anterior chamber biomechanics	Higher technical complexity; lower reproducibility than rigid systems	Displaced volume; pressure; recovery time
Spring-eye model [22]	Direct measurement of displaced volume using calibrated springs; enables inter-platform comparisons	Artificial model; does not fully reproduce biological tissue behavior	Surge volume; surge duration (ms)
Mechanical model of ocular compliance [14,22]	Mathematical simulation of human pressure–volume relationships; highly repeatable	Lacks biological variability; relies on modeled assumptions	Exact volume as a function of pressure (P–V curves)
Ex vivo (donor or banked eyes) [23,27,39]	Highest anatomical and tissue realism	Limited availability; inter-eye variability; tissue fatigue with repeated testing	Anterior chamber depth; lost aqueous volume

Abbreviations: ms: milliseconds; P–V curves: pressure–volume curves.

**Table 3:** Comparative results of occlusion break surge among phacoemulsification systems.

System	Surge Duration (Reported or Inferred from Experimental/Bench Data)	Surge Volume/Aqueous Loss *	Notes
Legacy (Alcon)	Not reported	Higher than Infiniti [51]	Early gravity-based system; high surge with 19G tip and high flow
Infiniti (Alcon)	Qualitative only (shorter than Legacy) [51]	~18% lower than Legacy [51]	ABS and low-compliance tubing reduce surge
Millennium (B&L)	Qualitative only (variable, mode-dependent) [51]	Lower in peristaltic mode [51]	Venturi mode associated with maximum surge
Sovereign (AMO)	Qualitative only (shorter with Cruise Control) [51]	Reduced with Cruise Control [51]	ABS + CC improve safety
Constellation (Alcon)	Qualitative only (variable) [55]	Higher than Centurion; comparable to Stellaris/EVA [55]	Designed primarily for vitreoretinal surgery; less efficient in surge control
Stellaris PC (B&L)	Not reported	67–163 µL (27–65% of AC volume) [46,47]	Higher surge among modern platforms
Whitestar Signature (J&J)	Not reported	30–103 µL (12–41% of AC volume) [52]	Intermediate performance; better than Stellaris
EVA (DORC)	Not reported.	47–165 µL (19–66% of AC volume) [55]	High system compliance; large surge values
Oertli OS4	Qualitative only (variable)	Intermediate values; higher than Centurion but lower than older systems [56,57]	No Active/Adaptive fluidics; stable at mid-range settings
Oertli Faros	Not reported	Surge dependent on aspiration mode [58,59,60]	Compact system; lacks advanced surge mitigation
Oertli CataRhex 3	Not reported	Higher than OS4/Faros [40,44]	Portable platform; limited surge control
Centurion (Alcon)	Reported (bench model; 268–1590 ms; IOP-dependent) [40].	17–77 µL (7–31% of AC volume) [40,44]	Active Fluidics improves chamber stability
Centurion + Active Sentry (Alcon)	Qualitative only (shorter; rapid recovery) [40,56].	≤74.7 µL; (≤30% of AC volume) [40,56]	Handpiece-integrated sensor vents vacuum before collapse
Quatera 700 (Zeiss)	Reported (221–471 ms) [56].	Slightly higher peak volume than Centurion (spring-eye model) [22]	Excellent pressure recovery; efficient leak compensation
Legion (Alcon)	Reported (70–90 ms [33]	≈70 µL (similar to Signature Pro) [33]	New-generation system; comparable to modern platforms
Unity VCS (next generation)	Reported (bench/preliminary; ~200–300 ms) [33]	<60 µL (<20% of AC volume) preliminary (manufacturer technical data) [33]	Predictive infusion; physiologic IOP operation; pending independent validation

Abbreviations. ABS: Alcon: Alcon, (Fort Worth, TX, USA); Aspiration Bypass System; AC: Anterior chamber; B&L: Bausch & Lomb (Bridgewater, NJ, USA); CC: Cruise Control; DORC: DORC (Zuidland, The Netherlands); J&J: Johnson & Johnson Vision, (Jacksonville, FL, USA); ms: milliseconds; µL: micro-liters; Oertli: Oertli Instrumente AG, (Berneck, Switzerland); VCS: Vision Control System; Zeiss: Carl Zeiss Meditec AG, (Jena, Germany). * Percentages are normalized to a reference anterior chamber volume of approximately 250 µL in a phakic adult eye; actual values may vary with axial length and anterior segment anatomy.

## Data Availability

Not applicable.

## Abbreviations

The following abbreviations are used in this manuscript:

ABS	Aspiration Bypass System
AC	Anterior chamber
ACV	Anterior chamber volume
BSS	Balanced salt solution
B&L	Bausch and Lomb
CCF	Centrally Controlled Fluidic
CC	Cruise Control
IOP	Intraocular Pressure
J&J	Johnson & Johnson
ms	milliseconds
µL	Micro-liters
OCT	Optical Coherence Tomography
OVDs	Ophthalmic viscosurgical devices
P–V	Pressure–Volume
PVD	posterior vitreous detachment
VCS	Vision Control System
